# Supplementary material for: Content-rich biological network constructed by mining PubMed abstracts
Source: BMC Bioinformatics. 2004 Oct 8;5:147. doi: 10.1186/1471-2105-5-147 (PMC528731; doi:10.1186/1471-2105-5-147)
Supplement: Additional File 2 — The original results of the above study (non-essential files are deleted to keep the file size under the limit set by BMC bioinformatics). [file 1471-2105-5-147-S2.bz2 › chilibotAdditionalFile2/dip40/290ID1833185E1120/html/CDC2_CCNB1.html]

 


 **CDC2** and **CCNB1** 
  
Found 825 abstracts in PubMed, retrieved 40.  
 

 What does Google say? 
 PDF only 
| .edu only 

---

**Interactive relationship** (e.g. stimulation, inhibition, etc)

**Inhibitory relationship**- Both down regulation of cell cycle regulators p34cdc2  [ **CDC2** ] , cyclin B1  [ **CCNB1** ]  and cdc25C and decreased association between p34cdc2  [ **CDC2** ]  and cyclin B1  [ **CCNB1** ]  were found to be associated with the activity of  **cdc2**  kinase abated after the H. pylori infection.  Ref: 12678330 Scand J Gastroenterol, 2003
- 3 1.5 mM caffeine that relieves cells from G 2 M arrest also inhibits As 2 O 3 induced Deltavarphi m collapse and apoptosis, 4 1.0 & ; mgr ; M As 2 O 3 increases the expression of both cyclin B  [ **CCNB1** ]  1 and hCDC20 whereas it inhibits Tyr15 phosphorylation of p34  **cdc2** .  Ref: 12835721 Leukemia< MedlineTA>Leukemia, 2003
- Thus, the impairment in cell cycle progression could not be explained by a lack of cyclin B1  [ **CCNB1** ] , or the ability of  **Cdc2**  and cyclin B1  [ **CCNB1** ]  to associate, but instead resulted, at least in part, from impaired  **Cdc2**  activity.  Ref: 12883482 Hepatology, 2003
- The addition of butyrolactone I, which is an inhibitor of CDK1  [ **CDC2** ]  and CDK2, to the p53 negative cells reduced the floating round cell population and induced the disappearance of cyclin B1  [ **CCNB1** ] .  Ref: 12646262 Arch Biochem Biophys, 2003
**Neutral relationship**- Immunoblot analysis revealed that ethanol metabolism had little affect on total  **Cdc2**  content in these cells, but resulted in the accumulation of up to 20 times the amount of cyclin B1  [ **CCNB1** ] , indicating that cyclin B1  [ **CCNB1** ]  was available for formation of  **Cdc2**  cyclin B1  [ **CCNB1** ]  complexes.  Ref: 12883482 Hepatology, 2003
- **Cdc2**  regulation also involves positive feedback, with active  **Cdc2**  cyclin B  [ **CCNB1** ]  stimulating its activator Cdc25 refs 5 7 and inactivating its inhibitors Wee1 and Myt1 refs 8 11.  Ref: 12629549 Nat Cell Biol, 2003
- **Cdc2**  is positively regulated by association with cyclin B1  [ **CCNB1** ] , and negatively regulated by phosphorylation of amino acids Thr14 and Tyr15.  Ref: 12883482 Hepatology, 2003
- Progression through mitosis occurs because cyclin B  [ **CCNB1** ]   **Cdc2**  activation induces the anaphase promoting complex APC to cause cyclin B  [ **CCNB1** ]  destruction and mitotic exit.  Ref: 12791267 Dev Cell, 2003
- These dynamic changes in cyclin B1  [ **CCNB1** ]   **Cdc2**  localization are regulated by phosphorylation of four serines within a region of cyclin B1  [ **CCNB1** ]  known as the cytoplasmic retention sequence CRS.  Ref: 12612056 Mol Cancer Res, 2003
- Consistent with these observations, CDK1  [ **CDC2** ]  activity was maintained at high levels, along with stable cyclin B1  [ **CCNB1** ] , in p53 negative cells.  Ref: 12646262 Arch Biochem Biophys, 2003

**Non-interactive relationship** (e.g. studied together, co-existance, homology, etc.)

- These results suggest that the RRASK motif in Xenopus cyclin B2 plays an important role in defining the substrate specificity of the cyclin B  [ **CCNB1** ]   **Cdc2**  complex.  Ref: 12754270 J Biol Chem, 2003
- The cyclin B1  [ **CCNB1** ]   **Cdc2**  complex regulates many of the dramatic cellular rearrangements observed at mitosis.  Ref: 12612056 Mol Cancer Res, 2003
- Finally, forced expression of the NF YA protein in cells committed to differentiate leads to an impairment in the down regulation of cyclin A, cyclin B1  [ **CCNB1** ] , and cdk1  [ **CDC2** ]  expression and is accompanied by a delay in myogenin expression.  Ref: 12857858 Mol Biol Cell, 2003
- Co immunoprecipitation revealed that 6 times more  **Cdc2**  cyclin B1  [ **CCNB1** ]  complexes were present in the ethanol treated cells compared with the controls.  Ref: 12883482 Hepatology, 2003
- Earlier we showed that U L 13 and ICP22 mediate the stabilization of  **cdc2**  and the replacement of its cellular partner, cyclin B  [ **CCNB1** ] , with the viral DNA polymerase processivity factor U L 42.  Ref: 12665617 Proc Natl Acad Sci U S A, 2003
- This was associated with earlier expression of the cell cycle promoting cyclin A2, cyclin E, cyclin B1  [ **CCNB1** ] , cyclin F and cyclin dependent kinase 1  [ **CDC2** ]  Cdk 1  [ **CDC2** ]  genes and diminished protein levels of Cdk  [ **CDC2** ]  inhibitor p21Cip1.  Ref: 12867420 J Biol ChemJ Biol Chem, 2003
- For example, in frog egg extracts, Cdk1  [ **CDC2** ]  cyclin B  [ **CCNB1** ]  catalyzes entry into mitosis but cannot trigger DNA replication.  Ref: 12738867 Science, 2003
- HEI10 is itself a specific in vitro substrate of purified cyclin B  [ **CCNB1** ]   **cdc2** , with a TPVR motif as primary phosphorylation site.  Ref: 12612082 Mol Cell Biol, 2003
- Monensin decreased the levels of CDK2, CDK6,  **cdc2** , cyclin A, cyclin B1  [ **CCNB1** ] , cyclin D1 and cyclin E proteins but did not alter CDK4 protein.  Ref: 12792794 Int J Oncol, 2003
- cyclins accumulate and produce active mitotic  **Cdc2**  cyclin B  [ **CCNB1** ] .  Ref: 12629549 Nat Cell Biol, 2003
- Increased levels of cyclin B1  [ **CCNB1** ]  and enhanced activity of its associated cyclin dependent kinase  **cdc2**  became apparent during the clonal evolution of p210 bcr abl transduced 32D cell clones lacking p53.  Ref: 12801837 Haematologica, 2003
- In order to understand the mechanism s of tumor promotion by MY and MG, we have studied the levels of PCNA, a marker of cell proliferation and cell cycle regulatory proteins, cyclin D1, and its associated kinase, cdk4, cyclin B1  [ **CCNB1** ] , and associated kinase,  **cdc2** .  Ref: 12616621 Teratog Carcinog Mutagen, 2003
- Positive staining for cyclin D1, cyclin E, cyclin A, cyclin B1  [ **CCNB1** ] , cdk2, cdk4,  **cdc2** , p53, p21, and p27 was observed in 63%, 66%, 31%, 32%, 51%, 77%, 71%, 43%, 35%, and 60% of the 82 carcinomas, respectively.  Ref: 12792921 Hum Pathol, 2003
- Moreover, cDNA array analysis showed that PC SPES caused up regulation of p21 WAF1 CIP1 and decreased expression of cyclin B  [ **CCNB1** ] , Nedd8,  **cdc2** , skp1, PCNA, MAD2L1, cyclin H, CKS2, E2F, Rbx1, MCM2, MCM5, Mpp2, Cullin Cul4A, Cks1p9 and McM7, which are involved in cell cycle progression.  Ref: 12695690 Cell Cycle< MedlineTA>Cell Cycle, 2003
- Activation of cyclin B  [ **CCNB1** ]   **Cdc2**  is an absolute requirement for entry into mitosis, but other protein kinase pathways that also have mitotic functions are activated during G 2 M progression.  Ref: 12609978 J Biol Chem, 2003
- The gene expression of cyclin B  [ **CCNB1** ]  and p34cdc2  [ **CDC2** ]  was increased after the 1 day treatment, reverted to the control level at 2 and 12 weeks of treatment, and was remarkably increased after the 52 week treatment.  Ref: 12841865 Cancer Sci, 2003
- Here, we show that Cdk1  [ **CDC2** ]  cyclin B1  [ **CCNB1** ]  has cryptic S phase promoting abilities that can be unmasked by relocating it from the cytoplasm to the nucleus and moderately stimulating its activity.  Ref: 12738867 Science, 2003
- p21 Waf1 Cip1 exerts a key role in driving this G 2 exit both by inhibiting cyclin B1  [ **CCNB1** ]  Cdk1  [ **CDC2** ]  and cyclin A Cdk1  [ **CDC2** ]  2 complexes, which control G 2 M progression, and by blocking the phosphorylation of pRb family proteins.  Ref: 12881433 EMBO JEMBO J, 2003
- Western and Northern blot hybridization showed an increased expression of both cyclin D1 and its associated kinase cdk4, and cyclin B1  [ **CCNB1** ]  and its associated kinase  **cdc2** , in livers of rats administered MY and MG after administration of DEN as compared to untreated or DEN controls.  Ref: 12616621 Teratog Carcinog Mutagen, 2003
- The mechanism of this uncoupling involves the cyclin B  [ **CCNB1** ]   **Cdc2**  dependent proteolytic cleavage of the N terminal ERK binding domain of MEK1 and the phosphorylation of Thr 286.  Ref: 12609978 J Biol Chem, 2003
- The interphase cytoplasmic location of cyclin B1  [ **CCNB1** ]   **Cdc2**  reflects continuous, albeit slow, nuclear import and much more rapid nuclear export.  Ref: 12612056 Mol Cancer Res, 2003
- Doxorubicin treatment did not alter cyclin B  [ **CCNB1** ]  or  **cdc2**  protein level, which is known as the essential component of G2 M transition.  Ref: 12579337 Int J Mol Med, 2003
- A stronger G2 M arrest by these combinations was accompanied by a substantial decrease in the levels of  **cdc2** , cyclin B1  [ **CCNB1** ]  and cdc25C.  Ref: 12866029 Int J CancerInt J Cancer, 2003
- 2 ME treatment results in phosphorylation of cdc25C without any significant alterations in the expression of cyclin B1  [ **CCNB1** ]  or p34cdc2  [ **CDC2** ] .  Ref: 12584169 Carcinogenesis, 2003
- We show that this conserved sequence in Xenopus cyclin B2, termed the RRASK motif, is required for the substrate recognition by the cyclin B  [ **CCNB1** ]   **Cdc2**  complex of Cdc25C.  Ref: 12754270 J Biol Chem, 2003
- These results suggest that the RRASK motif in Xenopus cyclin B2 plays an important role in defining the substrate specificity of the cyclin B  [ **CCNB1** ]   **Cdc2**  complex.  Ref: 12654907 J Biol Chem< MedlineTA>J Biol Chem, 2003
- The archival slides were incubated overnight at 4 degrees C using mouse anti human recombinant cyclin B1  [ **CCNB1** ]  polyclonal antibody or mouse anti Xenopus p34  **cdc2**  monoclonal antibody for immunohistochemistry Santa Cruz Biotech, Santa Cruz, CA.  Ref: 12821336 Gynecol Oncol, 2003
- When we examined the effects of this drug on ACHN cells, monensin decreased the levels of CDK2, CDK6,  **cdc2** , cyclin A and cyclin B1  [ **CCNB1** ]  proteins.  Ref: 12632079 Int J Oncol, 2003
- This genistein induced G2 M arrest in vitro was associated with a significant increase in the protein expression of phosphorylated p34  **cdc2**  and of cyclin B1  [ **CCNB1** ] .  Ref: 12668277 Cancer Lett, 2003
- Our results show that the mRNA and protein levels of the G 2 M regulators  **cdc2** , cyclin B1  [ **CCNB1** ]  and cdc25C are down regulated in MCF7TDX cells, while unaffected in MCF7 cells.  Ref: 12853888 Anticancer Drugs, 2003
- Flavopiridol treatment of docetaxel treated cells enhances the exit from mitosis with a more rapid decrease in mitotic markers including MPM 2 labeling and cyclin B1  [ **CCNB1** ]   **cdc2**  kinase activity.  Ref: 12813134 Mol Cancer Ther< MedlineTA>Mol Cancer Ther, 2003
- Degradation of Tome 1 during G1 allows for wee 1 accumulation during interphase, thereby providing a critical link between the APC and SCF pathways in regulation of cdk1  [ **CDC2** ]  cyclin B  [ **CCNB1** ]  activity and thus mitotic entry and exit.  Ref: 12679038 Cell, 2003
- Tome 1 is a cytosolic protein required for proper activation of cdk1  [ **CDC2** ]  cyclin B  [ **CCNB1** ]  and mitotic entry.  Ref: 12679038 Cell, 2003
- cyclin D1, cyclin B  [ **CCNB1** ] , and p34cdc2  [ **CDC2** ]  were examined by RT PCR.  Ref: 12841865 Cancer Sci, 2003
- Entry into mitosis requires the activation of cdk1  [ **CDC2** ]  cyclin B  [ **CCNB1** ] , while mitotic exit is achieved when the same kinase activity decreases, as cyclin B  [ **CCNB1** ]  is degraded.  Ref: 12679038 Cell, 2003
- These results demonstrate that cyclin B  [ **CCNB1** ]   **Cdc2**  activity regulates signaling through the MAPK pathway in mitosis.  Ref: 12609978 J Biol Chem, 2003
- Deregulated activity of the cyclin B1  [ **CCNB1** ]   **cdc2**  complex may be involved in the loss of temporal co ordination of mitotic events and further free the barrier to genomic instability of CML clonal myeloid progenitors lacking p53.  Ref: 12801837 Haematologica, 2003
- Furthermore, cyclin A CDK1  [ **CDC2** ]  2, cyclin B  [ **CCNB1** ]  CDK1  [ **CDC2** ]  2 and cyclin E CDK2 complexes can phosphorylate multiple p73 isoforms in vitro at threonine 86 T86.  Ref: 12676926 J Biol Chem< MedlineTA>J Biol Chem, 2003
- Collectively, these data demonstrate a new role for Erk in mitotic regulation, identify the Ser 101 directed kinase, and provide a picture of cyclin B1  [ **CCNB1** ]   **Cdc2**  regulation by the combinatorial action of distinct kinases.  Ref: 12612056 Mol Cancer Res, 2003
- We have previously found that the overexpression of p53 causes G 2 arrest and represses the synthesis of cyclin dependent kinase 1  [ **CDC2** ]  and cyclin B1  [ **CCNB1** ] , two proteins required for cells to traverse from G 2 into M.  Ref: 12874009 Cancer Res, 2003
- Two hypotheses can explain this observation Either Cdk1  [ **CDC2** ]  cyclin B  [ **CCNB1** ]  fails to recognize the key substrates of its S phase promoting counterparts, or its activity is somehow regulated to prevent it from activating DNA synthesis.  Ref: 12738867 Science, 2003
- We confirm that the response of  **Cdc2**  to non degradable cyclin B  [ **CCNB1** ]  is temporally abrupt and switch like, as would be expected if  **Cdc2**  activation were bistable.  Ref: 12629549 Nat Cell Biol, 2003
- The increased sensitivity to etoposide induced G 2 M arrest was associated with disruption of cell cycle related events, including hyperphosphorylation of p34  **cdc2**  kinase, change in cdc25C expression and phosphorylation, and alteration in cyclin B1  [ **CCNB1** ]  expression.  Ref: 12569391 Br J Cancer, 2003
- Both immunoreactivities to cyclin B  [ **CCNB1** ]  and p34  **cdc2**  were strongly correlated with the HPV infection P = 0.0001 and the histological types P = 0.0001 between CIN and SCC, being strongly correlated with each other alpha 0.62954, P = 0.0001.  Ref: 12821336 Gynecol Oncol, 2003
- Cells exit mitosis in a specific time window without cytokinesis with a decrease in cyclin B1  [ **CCNB1** ]  cdc 2  [ **CDC2** ]  kinase activity and MPM 2 labeling.  Ref: 12813134 Mol Cancer Ther< MedlineTA>Mol Cancer Ther, 2003
- Genetic analysis of fission yeast and physiological studies of vertebrate and invertebrate oocytes showed that activation of cyclin B  [ **CCNB1** ]   **cdc2**  kinase triggers mitosis.  Ref: 12836412 Med Sci Paris , 2003
- To ensure that cyclin B  [ **CCNB1** ]   **Cdc2**  does not prematurely activate the APC in early mitosis, there must be a mechanism delaying APC activation.  Ref: 12791267 Dev Cell, 2003
- The RRASK Motif in Xenopus Cyclin B2 Is Required for the Substrate Recognition of Cdc25C by the Cyclin B  [ **CCNB1** ]   **Cdc2**  Complex.  Ref: 12754270 J Biol Chem, 2003
- In contrast, pretreatment with flavopiridol prevents cells from entering mitosis by inhibiting cyclin B1  [ **CCNB1** ]  cdc 2  [ **CDC2** ]  kinase activity, thus antagonizing the docetaxel effect.  Ref: 12813134 Mol Cancer Ther< MedlineTA>Mol Cancer Ther, 2003
- The RRASK motif in Xenopus cyclin B2 is required for the substrate recognition of Cdc25C by the cyclin B  [ **CCNB1** ]   **Cdc2**  complex.  Ref: 12654907 J Biol Chem< MedlineTA>J Biol Chem, 2003
- In the early embryonic cell cycle,  **Cdc2**  cyclin B  [ **CCNB1** ]  functions like an autonomous oscillator, whose robust biochemical rhythm continues even when DNA replication or mitosis is blocked.  Ref: 12629549 Nat Cell Biol, 2003
- We show that this conserved sequence in Xenopus cyclin B2, termed the RRASK motif, is required for the substrate recognition by the cyclin B  [ **CCNB1** ]   **Cdc2**  complex of Cdc25C.  Ref: 12654907 J Biol Chem< MedlineTA>J Biol Chem, 2003
- The effect of STP on cell cycle arrest induced by X irradiation was studied in two cell lines by using flow cytometry, Western Blotting was performed to indicate the changes of cyclin B1  [ **CCNB1** ]  and  **cdc2**  protein levels.  Ref: 12579796 Yao Xue Xue Bao, 2002
